# Chinese herbal compound Huangqin Qingrechubi capsule reduces lipid metabolism disorder and inflammatory response in gouty arthritis *via* the LncRNA H19/APN/PI3K/AKT cascade

**DOI:** 10.1080/13880209.2023.2191641

**Published:** 2023-03-30

**Authors:** Xianheng Zhang, Jian Liu, Yanqiu Sun, Qin Zhou, Xiang Ding, Xiaolu Chen

**Affiliations:** a Department of Rheumatology and Immunology, First Affiliated Hospital of Anhui, University of Traditional Chinese Medicine, Hefei, China; b Institute of Rheumatology, Anhui University of Chinese Medicine, Hefei, China; c Anhui University of Traditional Chinese Medicine, Hefei, China

**Keywords:** Inflammation, adiponectin, fibroblast-like synoviocytes, mechanism of action

## Abstract

**Context:**

Gouty arthritis (GA) is a characteristically inflammatory disease often associated with lipid metabolism disorder. Huangqin Qingrechubi capsule (HQC) has been used for the treatment of GA.

**Objective:**

To explore the mechanism of HQC in the treatment of GA.

**Materials and methods:**

A total of 30 GA patients (GA group) and 30 healthy subjects [normal control (NC) group] were recruited. The GA group was treated with HQC (3.6 g/d) for 10 days. Lipid metabolism and inflammation indexes were detected. Five herbal names of HQC, or ‘gouty arthritis’, ‘hyperlipidemia’ and ‘inflammation’ were used as key words to search related databases for network pharmacological analysis. Subsequently, GA-fibroblast-like synoviocytes (FLSs) were stimulated with GA-peripheral blood mononuclear cells (PBMCs) (3:1) and treated with HQC drug-containing serum (20%). RT-qPCR, Western blot, and ELISA were conducted to further explore the mechanism of HQC in improving GA.

**Results:**

In clinical observation, HQC decreased the expression of lncRNA H19 and IL-1β, and increased the expression of adiponectin (APN) and IL-4 in the GA group (about half). Through network pharmacology, the PI3K/AKT signaling pathway was identified. Cell experiments showed that HQC treatment reduced the viability of GA-FLSs (49.61%), up-regulated the expression of IL-4 (155.18%), IL-10 (165.13%), and APN (31.24%), and down-regulated the expression of lncRNA H19 (33.70%), IL-1β (64.70%), TNF-α (78.32%), p-PI3K (48.80%), and p-AKT (53.48%).

**Discussion and conclusions:**

HQC improved lipid metabolism disorder and inflammatory response of GA by regulating the lncRNA H19/APN/PI3K/AKT. Maintaining the stability of lipid metabolism may be an effective way to alleviate GA.

## Introduction

Gouty arthritis (GA) is an inflammatory condition caused by the deposition of monosodium urate crystals in joints and periarticular tissues, commonly manifesting as severe joint pain (Diaz-Torne et al. 2021). As of 2016, the global prevalence of GA has reached 1–6.8%, with an annual incidence of 0.58–2.89 per 1000 people, showing an ascending trend year by year (Dehlin et al. 2020). An acute attack of joint pain not only compromises the life quality of GA patients but also imposes heavy burdens on the family and social health medical system (Schlesinger and Thiele 2010). In addition to the characteristic inflammatory response, the role of lipid metabolism disorder in the progression of GA cannot be ignored. Abnormal lipid accumulation can promote inflammatory response and oxidative stress in obese patients, leading to a variety of chronic inflammatory diseases (Fernández-Sánchez et al. 2011). A previous study of 1412 GA patients has found that more than half of GA patients suffer from lipid metabolism disorder (Zhang et al. 2020). Therefore, maintaining normal lipid metabolism is of great significance for the treatment of GA.

Long non-coding RNAs (lncRNAs), a group of non-coding RNAs with more than 200 nucleotides, mediate gene expression at the transcriptional, post-transcriptional, translational, and post-translational levels. LncRNAs are associated to a variety of biological functions (Marques-Rocha et al. 2015; Marchese et al. 2017). The role of lncRNA dysregulation-mediated inflammation and homeostasis in metabolic disorders is rarely studied. The imprinted oncofetal lncRNA H19 is identified as a carcinogenic lncRNA in a variety of cancers (Shermane Lim et al. 2021). Intriguingly, lncRNA H19 has also recently become a new research hotspot in arthritic diseases. Emerging evidence has demonstrated the critical involvement of lncRNA H19 in the proliferation and inflammatory response of synovial fibroblasts during the process of rheumatoid arthritis and GA (Fu et al. 2021; Xue et al. 2022). Nevertheless, the specific mechanism of lncRNA H19 in GA has not been fully clarified.

The phosphatidylinositol 3-kinase (PI3K)/protein kinase B (AKT) pathway represents an endogenous signal transduction pathway in mammalian cells that controls angiogenesis, cell growth, cell proliferation, and cell metabolism (Morales-Ducret et al. 1992; Haagenson and Wu 2010). The abnormal activation of PI3K/AKT and mammalian target of the rapamycin (mTOR) network inhibits the autophagy of synovial fibroblasts, promotes the proliferation of synovial cells, and triggers the release of inflammatory factors, thereby accelerating the course of rheumatoid arthritis (Qu et al. 2016). In addition, PI3K/AKT is implicated in GA inflammatory response by mediating macrophage autophagy (Lou et al. 2022). Accumulating studies have also emphasized the decisive role of PI3K/AKT in adipogenesis and lipid accumulation, suggesting the involvement of PI3K/AKT in the regulation of lipid metabolism (Kwon et al. 2022; Xu et al. 2022).

Adipokines are adipose tissue-derived factors that act on distant target organs through blood circulation, participating in metabolic regulation. Adiponectin (APN) is one such factor secreted in large quantities in human plasma. APN can increase insulin sensitivity and enhance the hypoglycemic effect, representing a potential target for diabetes and atherosclerosis (Lei et al. 2021; Nielsen et al. 2021). With the deepening of research, the role of APN in inflammation is gradually recognized. APN can play an anti-inflammatory or pro-inflammatory role in different diseases (Choi et al. 2020). Since GA is an inflammatory disease accompanied by abnormal lipid metabolism, it is particularly necessary to explore the role of APN in the process of GA, but currently, there is a lack of relevant research.

Huangqin Qingrechubi capsule (HQC) (Anhui medicine No. ZL201110095718.X), a traditional Chinese medicine compound prepared by the First Affiliated Hospital of Anhui University of Traditional Chinese Medicine, is mainly composed of Radix Scutellariae (Chinese name Huang Qin; *Scutellariae baicalensis* Georgi [Lamiaceae]), Gardenia (Chinese name Zhi Zi; *Gardenia jasminoides* Ellis [Rubiaceae]), Clematis Chinensis (Chinese name Wei Ling Xian; *Clematis chinensis* Osbeck [Ranunculaceae]), Peach Seed (Chinese name Tao Ren; *Prunus persica* (L.) Batsch [Rosaceae]), Coix Seed (Chinese name Yi Yi Ren; *Coix lacryma-jobi* L.var.mayuen(Roman.) Stap [Gramineae]). HQC has shown notable efficacy and safety in the clinical treatment of GA (Wang et al. 2014). Proteomic studies have revealed that HQC can alleviate the inflammatory state and improve the anti-inflammatory ability of GA patients through specific proteins in macrophages such as interleukin-1 beta (IL-1β) and interleukin-8 (IL-8) (Sun et al. 2021). In addition, HQC can also up-regulate the level of phosphatase and tensin homolog (PTEN) protein and inhibit the activation of the PI3K/AKT signaling pathway, thus inhibiting the growth and inflammatory response of cardiomyocytes in GA (Sun et al. 2021). Our team has also conducted a retrospective study on 796 GA patients complicated with hyperlipidemia based on the data mining method and found that HQC can reduce the erythrocyte sedimentation rate (ESR) and triglyceride (TG) level in these patients (Zhang et al. 2021). However, its specific mechanism needs to be further explored.

In this study, we first explored the relationship between APN, lipid metabolism, and inflammation through clinical observation and observed the changes in lncRNA H19, APN, lipids, and inflammatory indicators after HQC treatment. Then, we determined the possible components and pathways of HQC-mediated inflammatory response and lipid metabolism in GA through network pharmacology. Finally, the possible mechanism of HQC regulating lipid metabolism and inhibiting GA inflammation was verified at the molecular level through cell experiments.

Our results confirmed that HQC up-regulated the expression of APN by mediating lncRNA H19 expression and inhibited the activation of the PI3K/AKT pathway, thereby alleviating GA inflammation and lipid metabolism imbalance. APN served as a key factor in regulating GA lipid metabolism and inflammation. These findings contribute to elucidate the pathogenesis of GA and provide an important theoretical basis for HQC treatment of GA.

## Materials and methods

### Subject recruitment and sample collection

This study recruited 30 GA patients who were admitted to the Rheumatology and Immunology Department of the First Affiliated Hospital of Anhui University of Traditional Chinese Medicine from December 2020 to April 2021. All the recruited GA subjects met the criteria guidelines for gout classification issued by the American College of Rheumatology/European League Against Rheumatology in 2015 (Neogi et al. 2015). This study was approved by the Ethics Committee of the First Affiliated Hospital of Anhui University of Traditional Chinese Medicine (Ethics number: 2021AH-27). All participants provided written informed consent.

GA subjects with the following conditions were excluded: (a) infections, (b) adolescents or pregnant women, (c) tumors, (d) rheumatoid arthritis, mandatory spondylitis, and other rheumatic diseases. All 30 GA patients received HQC treatment (1.2 g, oral, three times a day) in addition to routine treatment such as non-steroidal anti-inflammatory drugs (Celecoxib Capsules, 0.2 g, oral, once a day) for 10 d. In addition, 30 healthy subjects with no history of systemic inflammation or tumors were recruited from the Physical Examination Center of the First Affiliated Hospital as control. We extracted 5 mL of peripheral blood from each subject, and the serum sample was stored at −80 °C after centrifugation (1000 rpm at 37 °C for 10 min). The Ficoll density gradient method was used for the isolation of peripheral blood mononuclear cells (PBMCs). The clinical data of all subjects including age, gender, weight, course of the disease, hypersensitive C-reactive protein (hs-CRP), total cholesterol (TC), ESR, high-density lipoprotein cholesterol (HDL-C), TG, low-density lipoprotein cholesterol (LDL-C), blood uric acid (BUA), and visual analog scale (VAS) were collected.

### Cell culture and co-culture

Human primary fibroblast-like synoviocytes (FLSs) (RAB-iCell-s004) and human GA primary fibroblast-like synoviocytes (GA-FLSs) (HUM-iCELL-s036) isolated from the ankle joint were purchased from Saibaikang iCell Bioscience Inc (Shanghai, China) and cultured in RPMI-1640 medium containing 10% fetal bovine serum, 100 IU/mL penicillin, and 100 IU/mL streptomycin in a conventional cell culture environment. The medium was refreshed every 2–3 d. Upon reaching 80–90% confluence, the cells were rinsed with phosphate-buffered saline (PBS) twice and added with 0.25% trypsin, and the cell digestion was observed under the microscope. The trypsin was removed when the cells adhered to the wall loosely, and then, the complete medium was added. The cells were seeded into the new Petri dish at a ratio of 1:3 and cultured with an appropriate complete medium until the cell density reached 80–90%. In the transwell system, GA-PBMCs were placed in the apical chamber and GA-FLSs were placed in the basolateral chamber for 48 h. When the cell confluence reached 70–90%, the cells in each transwell chamber were removed.

### Reagent treatment and cell transfection

GA-FLSs were treated with YS-49 (10 μm) (HY-15477, MedChem Express, China), an activator of the PI3K/AKT pathway, for 24 h, and the expressions of the pathway-related proteins were detected (Chaussade et al. 2007). To overexpress lncRNA H19, the lncRNA H19 sequence was inserted into the pcDNA3.1 plasmid (GenePharma, Shanghai, China), and an empty pcDNA3.1 vector was also constructed. In addition, three small interfering RNA (si-RNA) sequences targeting lncRNA H19 were synthesized to silence lncRNA H19. Three small hairpin RNAs (shRNAs) with APN interference sequences were constructed with lentivirus as vectors (Table 1). GA-FLSs stimulated by GA-PBMCs were seeded into 6-well plates, and cell transfection was carried out when the cell confluence reached 60%-80%. Transfection efficiency was detected after 48 h.

**Table 1.** Primer sequences for RT-qPCR.

Target	Amplicon size(bp)	Primer sequence (5′–3′)
LncRNA H19	82	F:GCGGGTCTGTTTCTTTACTTCC R:TCTTTCATGTTGTGGGTTCTGG
β-actin	96	F:CCCTGGAGAAGAGCTACGAG R:GGAAGGAAGGCTGGAAGAGT
si-lncRNA H19 1#	NA	F:GCGGGUCUGUUUCUUUACUTT R:AGUAAAGAAACAGACCCGCTT
si-lncRNA H19 2#	NA	F:CCCGUCCCUUCUGAAUUUATT R:UAAAUUCAGAAGGGACGGGTT
si-lncRNA H19 3#	NA	F:CUGGACUCAUCAUCAAUAATT R:UUAUUGAUGAUGAGUCCAGTT
APN shRNA 1#	NA	TCGGGCACAGATCATTAATGA
APN shRNA 2#	NA	GCCCACCTGGAACTTGAAAGA
APN shRNA 3#	NA	CACCACCTTGGACCAAAGTAA

RT-qPCR: reverse transcription quantitative polymerase chain reaction; APN: adiponectin; F: forward; R: reverse; NA: not applicable.

### Enzyme-linked immunosorbent assay (ELISA)

The concentrations of cytokines in the serum or GA-FLSs supernatant were detected in line with the instruction of ELISA kits for interleukin-4 (IL-4) (JYM0142Hu), interleukin-10 (IL-10) (JYM0155Hu), IL-1β (JYM0083Hu), tumor necrosis factor-alpha (TNF-α) (JYM0110Hu), and APN (JYM1859Hu, Wuhan, China).

### Real-time quantitative polymerase chain reaction

The total RNA was isolated using the RNAiso Plus Reagent (TaKaRa, Japan). The PrimeScript™RT reagent Kit with gDNA Eraser (TaKaRa, Japan) was used to synthesize cDNAs, with the β-actin as the internal reference. The relative expressions of genes were calculated using the 2^−ΔΔC^_t_ method.

### Cell counting kit-8 (CCK-8) assay

The cell proliferation was detected using the CCK-8 assay kit (Bioss, China) as instructed. Firstly, 5 × 10^4^ GA-FLSs in the logarithmic phase were seeded into 96-well plates, transfected with the plasmid, and cultured for 24 h, with 3 duplicated wells in each group. Then, 10 µL CCK-8 solution was added to each well at 0, 24, 48, and 72 h. The absorbance value of each well was measured at the wavelength 450 nm after 1.5-h culture.

### Western blot

The total protein was extracted using radioimmunoprecipitation assay (RIPA) lysate, followed by protein quantification, sample loading, electrophoresis, and membrane transfer. The blots were blocked with 5% skim milk at room temperature for 2 h. After washing, the primary antibodies p-PI3K, p-AKT, and APN were added for incubation at 4 °C overnight. Then, these blots were washed and incubated with the secondary antibody (horseradish peroxidase-labeled) at a dilution ratio of 1:2 × 10^4^ for 1 h at room temperature. After rinsing 3 times, the protein contents were measured using the electrochemiluminescence (ECL) luminescence kit.

### Preparation of HQC drug-containing serum

A total of 20 specific pathogen-free male SD rats (weighing 180–220 g; Lscxk 2017-001, Laboratory Animal Center of Anhui Medical University) were randomly assigned to the normal group and HQC group after 1 week of adaptive feeding, with 10 rats in each group. HQC drug-containing serum was prepared according to the previous method (Wang et al. 2021). Briefly, HQC (360 mg/kg) was administered into rats by gavage (10 mL/kg). The control group was given the same volume of normal saline. All rats were given gavage twice a day for 3 consecutive days. One hour after the last administration, abdominal anesthesia was performed and blood was collected from the abdominal aorta, followed by centrifugation, filtration, sterilization, and storage at −80 °C for use. Animal experiments were reviewed and approved by the Animal Ethics Committee of the First Affiliated Hospital of Anhui University of Chinese Medicine (No. AHUCM- rats-2021022).

### Screening of HQC active ingredients and targets

Drug-likeness (DL) ≥ 0.18 and oral bioavailability (OB) ≥ 30% were set to obtain the active components of five traditional Chinese herbs contained in HQC through the Traditional Chinese Medicine Systems Pharmacology Database (TCMSP, https://tcmspw.com), and then, the corresponding protein targets of the active components were obtained. The protein type was set to ‘Homo sapiens’ and the protein-gene information was obtained from the UniProt database (https://www.uniprot.org/). After correction, the active ingredient-gene information was finally obtained.

### Gene acquisition related to GA, inflammation, and hyperlipidemia

‘Gouty arthritis’ or ‘arthritis, gouty’, ‘inflammation’, and ‘hyperlipidemia’ were selected as key words respectively to obtain disease-related genes from the Online Mendelian Inheritance in Man (OMIM, https://www.omim.org/), Gene Database (GeneCards, https://www.genecards.org/), DrugBank database (https://www.drugbank.ca/), Therapeutic Target Database (TTD, http://db.idrblab.net/ttd/), and DisGeNET database (https://www.disgenet.org/). The results obtained from the above databases were integrated and weighed to obtain genetic information related to GA inflammation and lipid metabolism.

### Underlying genes of HQC in treating GA inflammation and lipid metabolism and network construction of underlying genes-active ingredients

The target genes related to the active ingredients of HQC were intersected with the genes related to GA inflammation and lipid metabolism, and the underlying genes of HQC in treating GA inflammation and lipid metabolism were obtained. Then we imported the network information of active ingredients and underlying genes into the Cytoscape 3.7.2 software for network visualization.

### Protein-protein interaction (PPI) network construction and core target screening

The underlying genes obtained were imported into the BisoGenet plug-in of Cytoscape 3.7.2 software to construct a PPI network and the CytoNCA plug-in was used for topology analysis of central variables. Degree centrality (DC) ≥ 64 (2 times DC mean) was set for the first core target screening. Betweenness centrality (BC) ≥ 0.007 (mean) and closeness centrality (CC) ≥ 0.477 (mean) were set to obtain the final core target.

### Kyoto encyclopedia of genes and genomes (KEGG) and gene ontology (GO) analyses

The core target was introduced into Metascape (https://metascape.org/), and the species was selected as Homo sapiens. KEGG and GO enrichment analyses were performed at *p* < 0.01. The online data visualization platform (http://www.bioinformatics.com.cn/) was used for visualization.

### Statistical analysis

The GraphPad Prism 8.2 software (GraphPad Software, La Jolla, CA, USA) was used for statistical analysis and image rendering. The paired *t*-test or Kruskal–Wallis nonparametric test was used to test the significance of differences between the two groups, and the Chi-square test was used to compare the categorical variables. The Pearson correlation coefficient was adopted for correlation analysis. The data were expressed as means and standard deviation (SD) or quartile. The difference was statistically significant with a probability of *p*** **<** **0.05.

## Results

### Comparison of clinical data

**Figure 1.**
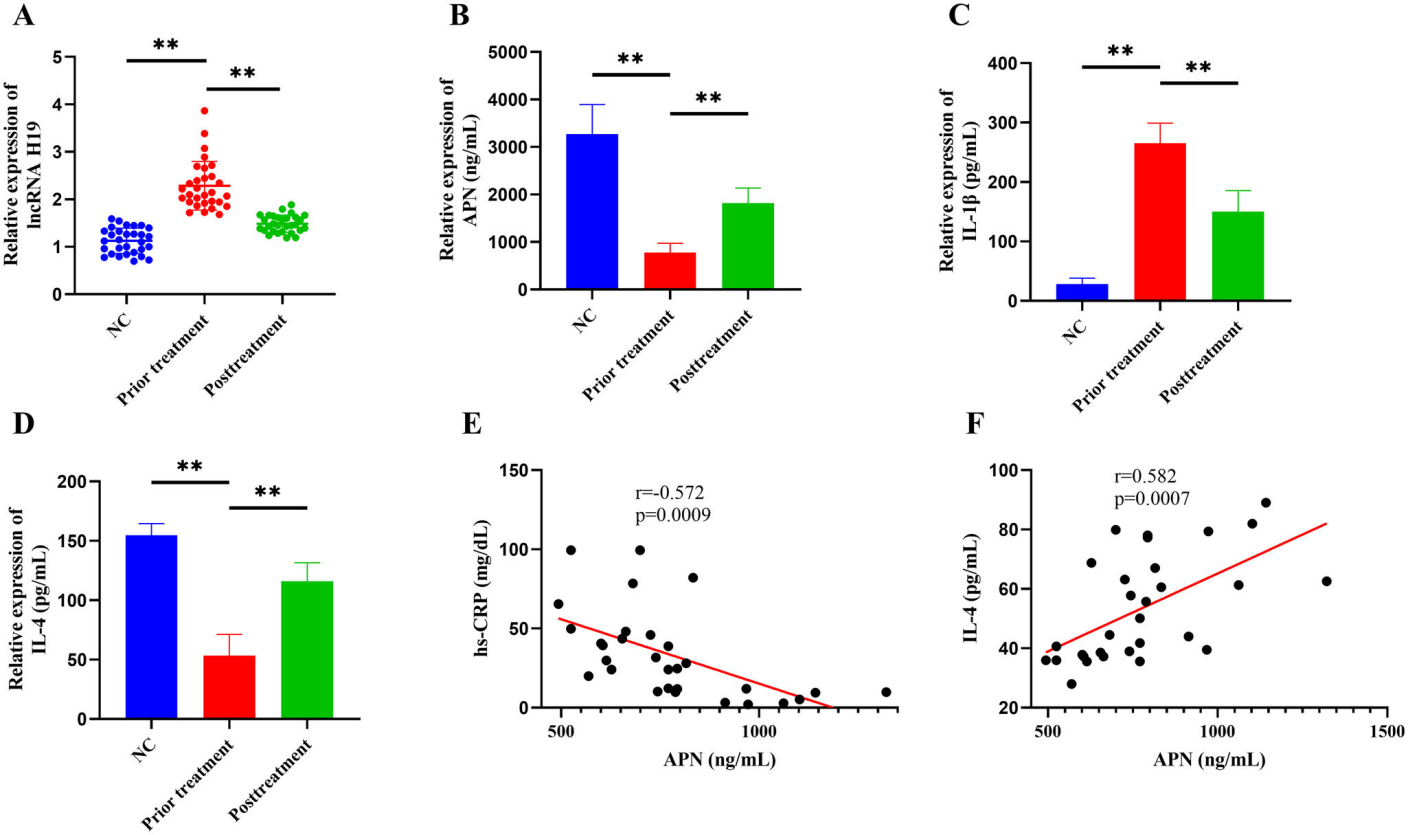
(A) The expression of long non-coding (lnc) RNA H19 in peripheral blood mononuclear cells (PBMCs) was detected by real-time quantitative polymerase chain reaction. (B–D) The expression of adiponectin (APN), interleukin-4 (IL-4), and interleukin-1 beta (IL-1β) in the supernatant of PBMCs was detected by enzyme-linked immunosorbent assay. (E) Spearman correlation analysis was used to explore the correlation between APN and hypersensitive C-reactive protein (hs-CRP). (F) Spearman correlation analysis was used to explore the correlation between APN and IL-4. **p < 0.01.

This study included 60 subjects, including 30 healthy subjects (23 males and 7 females) in the normal control (NC) group and 30 GA patients (25 males and 5 females) in the GA group. There was no statistical significance in age, weight, gender distribution, TC, and LDL-C between the two groups (*p*** **>** **0.05), but there were significant differences in hs-CRP, BUA, ESR, TG, and HDL-C (*p*** **<** **0.05) (Table 1). In the detection of PBMCs, we found that the GA group had significantly up-regulated expressions of lncRNA H19 and IL-1β but significantly down-regulated expressions of APN and IL-4 relative to the NC group (*p*** **<** **0.05) (Figure 1(A–D)). HQC treatment significantly decreased the expressions of lncRNA H19, hs-CRP, BUA, ESR, TG, IL-1β, and VAS but increased the expressions of APN, HDL-C, and IL-4 (*p*** **<** **0.05) (Table 2) (Figure 1(A–D)). In addition, correlation analysis revealed that APN was negatively correlated with hs-CRP but positively correlated with IL-4 (*p*≪0.05) (Figure 1(E,F)). These results suggested that GA patients exhibited lipid metabolism disorder and inflammatory response, and HQC might regulate GA inflammation and lipid metabolism through lncRNA H19 and APN.

**Table 2.** Baseline characteristics of the subjects.

Quantitative variables	NC (*n* = 30)	GA (*n* = 30)		*p*_1_ value	*p*_2_ value
		Prior treatment	Post treatment		
Gender, male/female, *n*	23/7	25/5	NA	0.519	NA
Age, y	59.50 (46.00, 74.25)	52.73 ± 13.94	NA	0.137	NA
Weight, kg	75.87 ± 8.30	76.00 (69.00, 81.25)	NA	0.965	NA
The course of the disease, y	NA	7.50 (3.50, 10.00)	NA	NA	NA
hs-CRP, mg/dL	5.05 (2.97, 12.00)	26.33 (10.12, 46.43)	2.75 (0.94, 10.60)	<0.001^a^	0.002^a^
ESR, mm/h	9.00 (5.90, 13.00)	32.17 (17.59, 59.00)	12.00 (5.00, 22.75)	<0.001^a^	<0.001^a^
TC, mmol/L	4.70 ± 0.77	4.94 ± 0.87	4.60 ± 0.95	0.254	0.162
TG, mmol/L	1.22 ± 0.41	1.86 (1.20, 2.16)	1.70 (1.13, 1.89)	0.002^a^	0.006^a^
HDL-C, mmol/L	1.07 (0.88, 1.27)	0.92 (0.82, 1.08)	1.30 ± 0.31	0.031^a^	<0.001^a^
LDL-C, mmol/L	2.74 ± 0.94	2.91 (2.53, 3.38)	3.06 (2.44, 3.58)	0.098	0.926
BUA, μmmol/L	323.30 ± 79.30	528.30 ± 135.63	394.03 ± 87.45	<0.001^a^	<0.001^a^
VAS	NA	4.53 ± 1.19	1.00 (0.50, 1.63)	NA	<0.001^a^

NC: normal control; GA: gouty arthritis; hs-CRP: hypersensitive C-reactive protein; ESR: erythrocyte sedimentation rate; TC: total cholesterol; TG: triglyceride; HDL-C: high-density lipoprotein cholesterol; LDL-C: low-density lipoprotein cholesterin; BUA: blood uric acid; VAS: visual analog scale; NA: not applicable.

Data are expressed as *n* or mean ± standard deviation; P1: NC vs. Prior treatment; P2: Prior treatment vs. Posttreatment.

^a^*p* < 0.05.

### Collection of HQC and disease target genes

**Figure 2.**
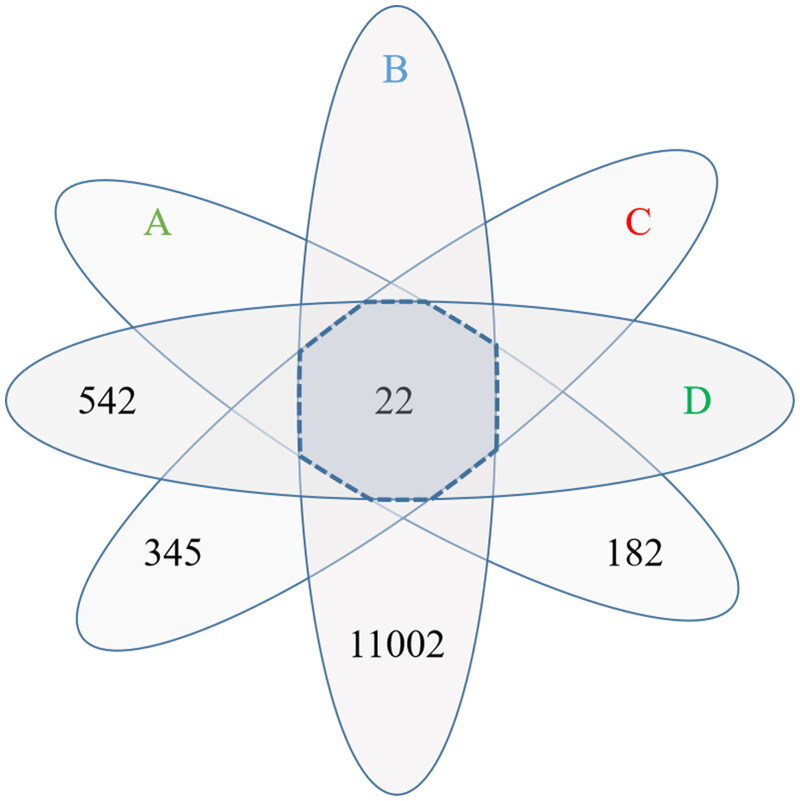
Venn diagram of drug target genes and disease target genes. (A) Huangqin Qingrechubi capsule, (B) inflammation, (C) hyperlipidemia, (D) gouty arthritis. The number around the petal is the number of target genes and the number in the center of the petal is the number of common target genes.

The active ingredients of five Chinese herbal medicines contained in HQC were collected through the TCMSP database, including 36 from radix scutellariae, 15 from gardenia, 7 from clematis chinensis, 23 from peach seed, and 9 from coix seed. After removing the duplicates, 78 active ingredients were obtained (Table 3). In addition, a total of 182 target genes were obtained after correction by the UniProt database. In the collection of disease target genes, there were 542 gout-related genes, 345 hyperlipidemia-related genes, and 11,002 inflammation-related genes. The target genes of HQC were intersected with the target genes of the three diseases, and 22 common drug-disease genes were finally obtained (Figure 2).

**Table 3.** Some active ingredient information of HQC.

TCM	MOL ID	Name of molecular	OB (%)	DL
radix scutellariae	MOL002934	Neobaicalein	104.34	0.44
	MOL002932	Panicolin	76.26	0.29
	MOL012246	5,7,4'-Trihydroxy-8-methoxyflavanone	74.24	0.26
	MOL002927	Skullcapflavone II	69.51	0.44
	MOL002911	2,6,2',4'-Tetrahydroxy-6'-methoxychaleone	69.04	0.22
	MOL002937	Dihydrooroxylin	66.06	0.23
gardenia	MOL004561	Sudan III	84.07	0.59
	MOL007245	3-Methylkempferol	60.16	0.26
	MOL003095	5-Hydroxy-7-methoxy-2-(3,4,5-trimethoxyphenyl)chromone	51.96	0.41
	MOL000098	Quercetin	46.43	0.28
	MOL009038	GBGB	45.58	0.83
	MOL001942	Isoimperatorin	45.46	0.23
clematis chinensis	MOL000449	Stigmasterol	43.83	0.76
	MOL005603	Heptyl phthalate	42.26	0.31
	MOL005594	ClematosideA'_qt	37.51	0.76
	MOL000358	beta-Sitosterol	36.91	0.75
	MOL005598	Embinin	33.91	0.73
	MOL002372	(6*Z*,10*E*,14*E*,18*E*)-2,6,10,15,19,23-hexamethyltetracosa-2,6,10,14,18,22-hexaene	33.55	0.42
Peach seed	MOL001371	Populoside_qt	108.89	0.20
	MOL001351	Gibberellin A44	101.61	0.54
	MOL001348	Gibberellin 17	94.64	0.49
	MOL001353	GA60	93.17	0.53
	MOL001349	4a-Formyl-7alpha-hydroxy-1-methyl-8-methylidene-4alpha,4beta-gibbane-1alpha,10beta-dicarboxylic acid	88.6	0.46
	MOL001344	GA122-isolactone	88.11	0.54
Coix seed	MOL000449	Stigmasterol	43.83	0.76
	MOL001323	Sitosterol alpha1	43.28	0.78
	MOL001494	Mandenol	42.00	0.19
	MOL000953	CLR	37.87	0.68
	MOL000359	Sitosterol	36.91	0.75
	MOL008121	2-Monoolein	34.23	0.29

HQC: Huangqin Qingrechubi capsule; TCM: traditional Chinese medicine; OB: oral bioavailability; DL: drug-likeness.

### PPI network construction and core target screening

**Figure 3.**
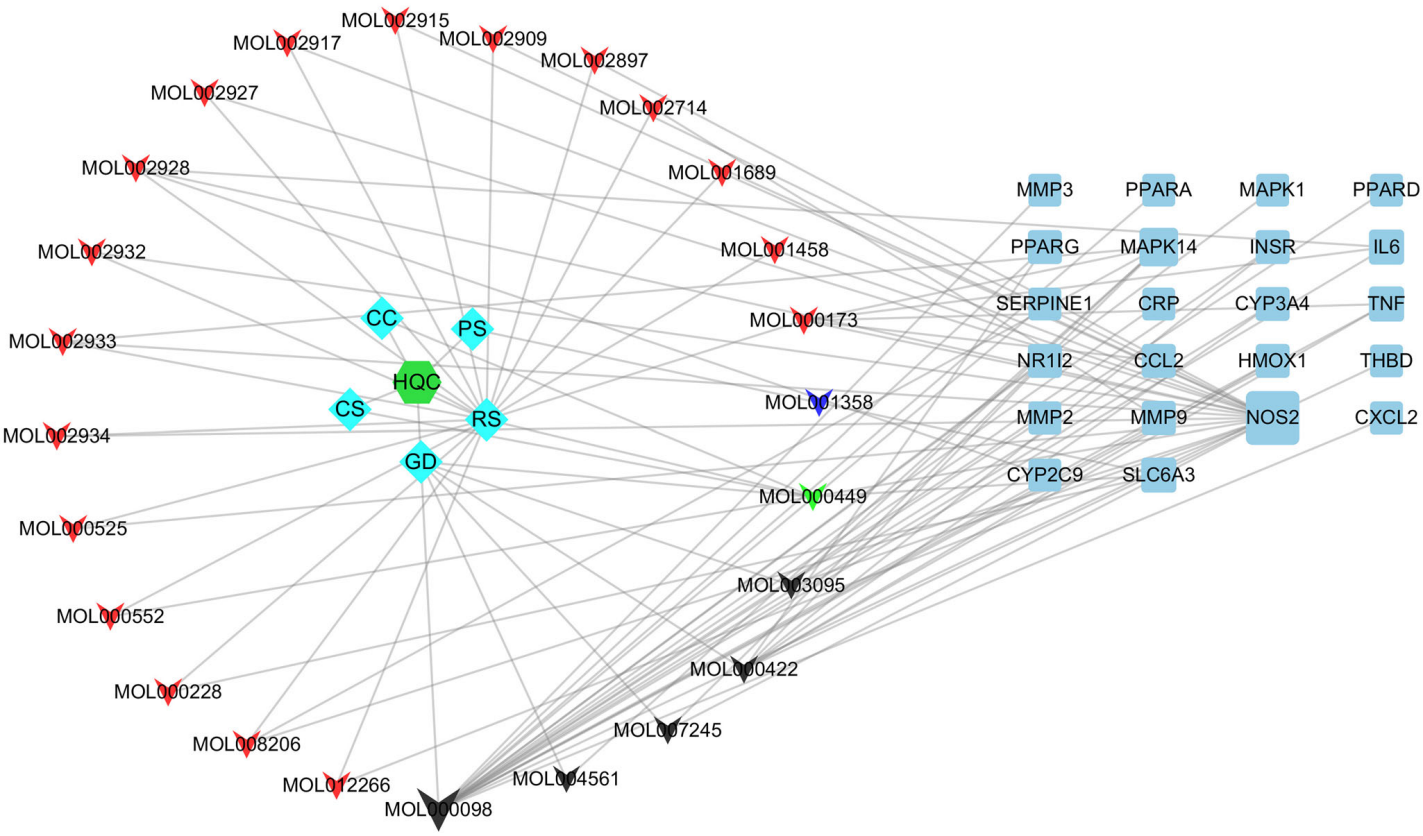
Drug-gene-gouty arthritis network diagram. The regular hexagon in the middle of the left side is HQC, which is the Huangqin Qingrechubi capsule. The diamond on the left represents the drug, where RS is radix scutellariae, GD is gardenia, CC is *Clematis chinensis*, PS is a peach seed, and CS is coix seed. The triangle on the left represents the active ingredient, in which red represents the active ingredient contained in RS, blue represents the active ingredient contained in PS, black represents the active ingredient contained in GD, and the green represents the active ingredient contained in RS, GD, CC, and CS. The right square is the target gene, and the larger the area, the higher the importance.

**Figure 4.**
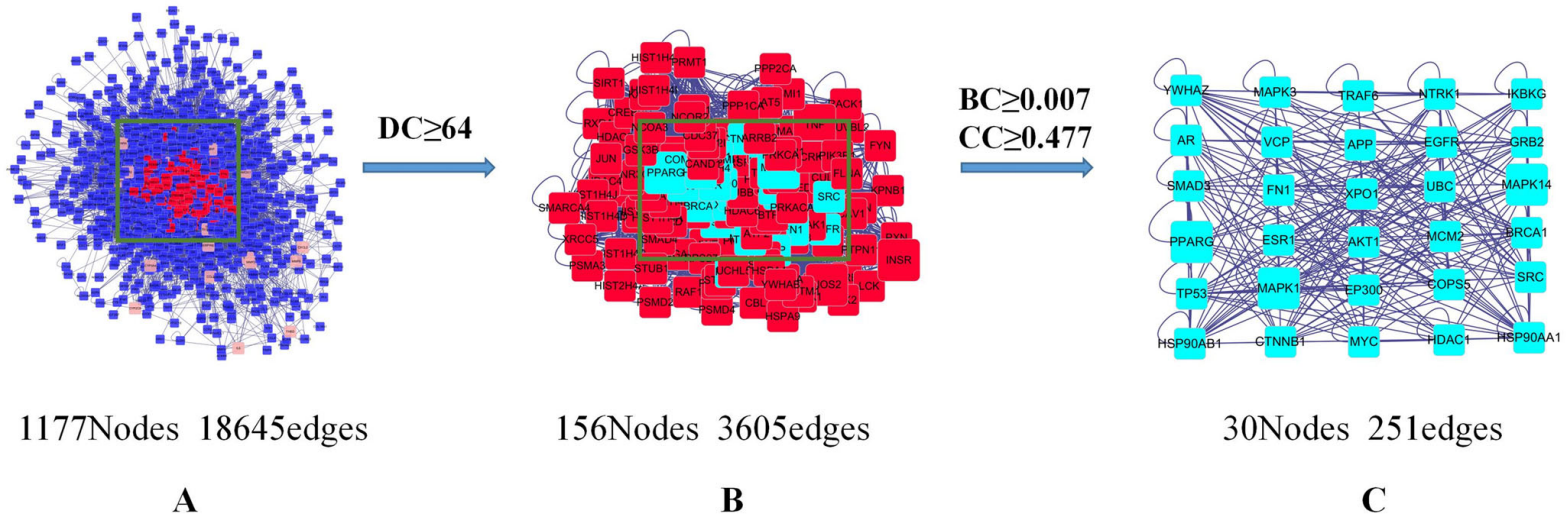
Core protein target screening map. (A) Based on 22 common genes and topology analysis, a protein-protein interaction (PPI) network with 1177 nodes and 18,645 edges was obtained. (B) A PPI network with 156 nodes and 3605 edges was obtained by setting degree centrality (DC) ≥ 64 (2 times the DC mean). (C) Betweenness centrality (BC) ≥ 0.007 (mean) and closeness centrality (CC) ≥ 0.477 (mean) were set to obtain a PPI network with 30 nodes and 251 edges. The 30 nodes are the core targets finally screened.

Based on the 22 common target genes, we constructed a network map of drug-active component-gene using the Cytoscape 3.7.2 software (Figure 3). Subsequently, we constructed a PPI network in the BisoGenet plug-in, and 30 core protein targets were screened out after topology analysis (Figure 4).

### GO and KEGG analyses

**Figure 5.**
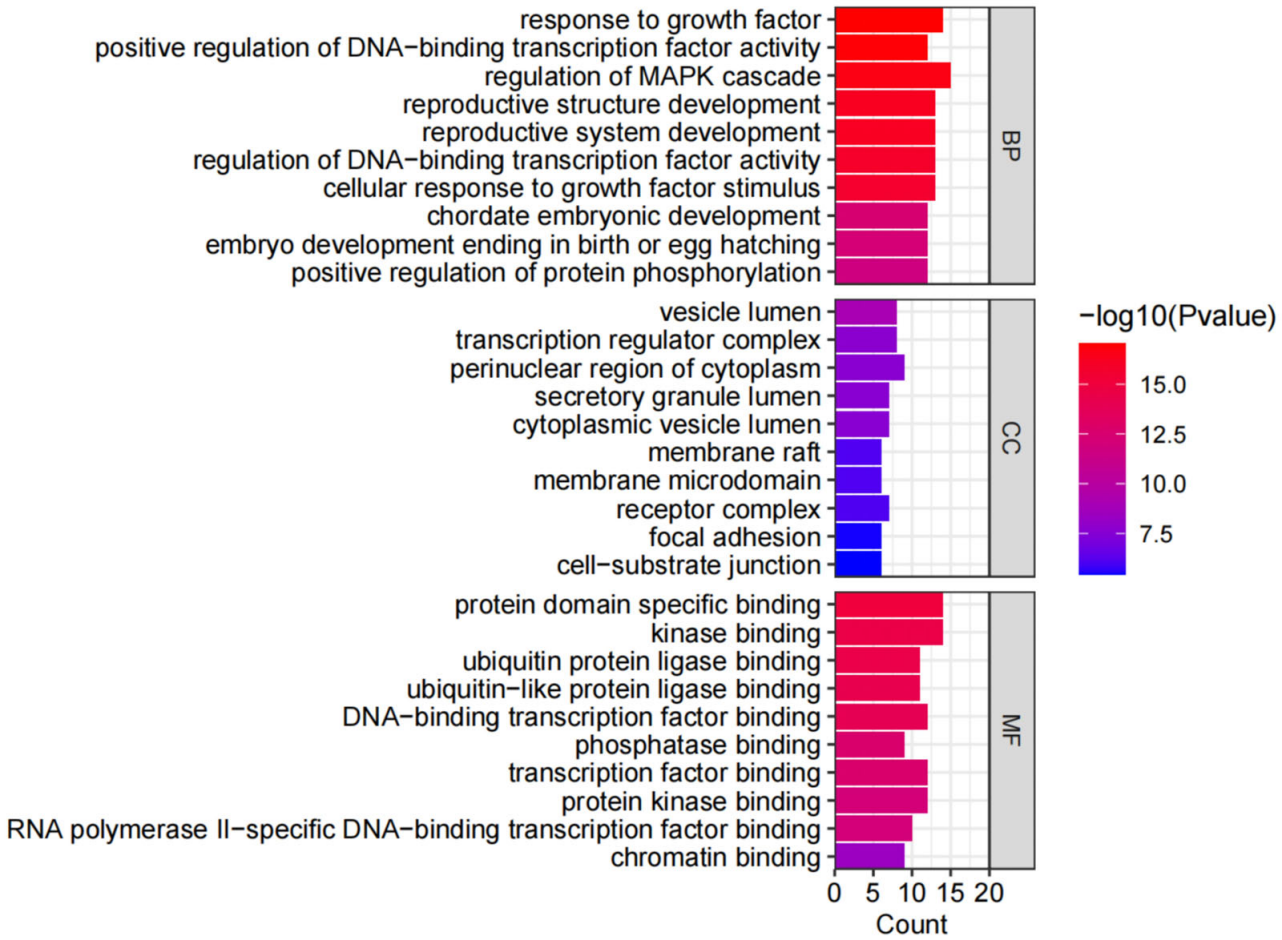
Gene ontology (GO) enrichment analysis. Biological process (BP), cellular component (CC), and molecular functions (MF) are enriched here. The ordinate is the project description, and the abscissa is the number of targets enriched. The color represents different *p* values, and the redder the color, the more significant it is.

**Figure 6.**
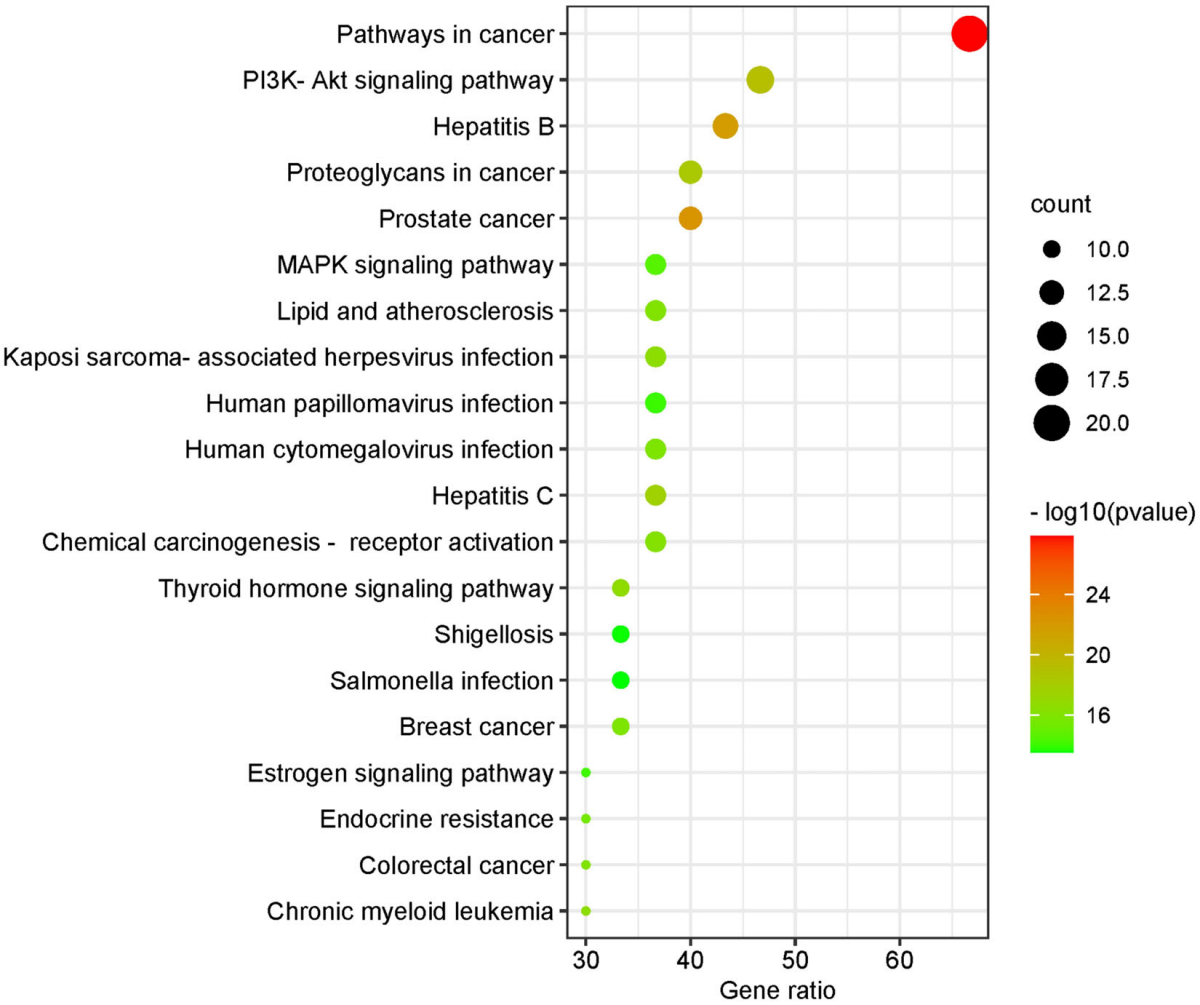
Kyoto encyclopedia of genes and genomes (KEGG) enrichment analysis. The ordinate is the project description, and the ordinate is the ratio of the number of enriched targets to the total number of targets. The color represents different *p* values, and the redder the color, the more significant it is.

GO analysis covered three domains: biological process (BP), cellular component (CC), and molecular function (MF). The results showed that genes were most significantly enriched in 716 BP terms (including response to growth factor, regulation of DNA-binding transcription factor activity, reproductive structure development, etc.), 57 CC terms (including the perinuclear region of cytoplasm, vesicle lumen, transcription regulator complex, etc.), and 70 MF terms (including protein domain specific binding, kinase binding, transcription factor binding, etc.). Due to a large number of terms, we only listed the terms with the top 10 enrichment targets in visualization (Figure 5). A total of 136 items were obtained in the KEGG analysis, and the top 10 pathways were visualized according to the number of enrichment targets (Figure 6). In addition to the first cancer-related pathway, the second PI3K/AKT signaling pathway also attracted our attention. We speculated that the PI3K/AKT signaling pathway played a key role in the treatment of GA lipid metabolism disorder and inflammation by HQC. So, we chose the PI3K/AKT signaling pathway for further cell experiments.

### Screening of co-culture concentration and time

**Figure 7.**
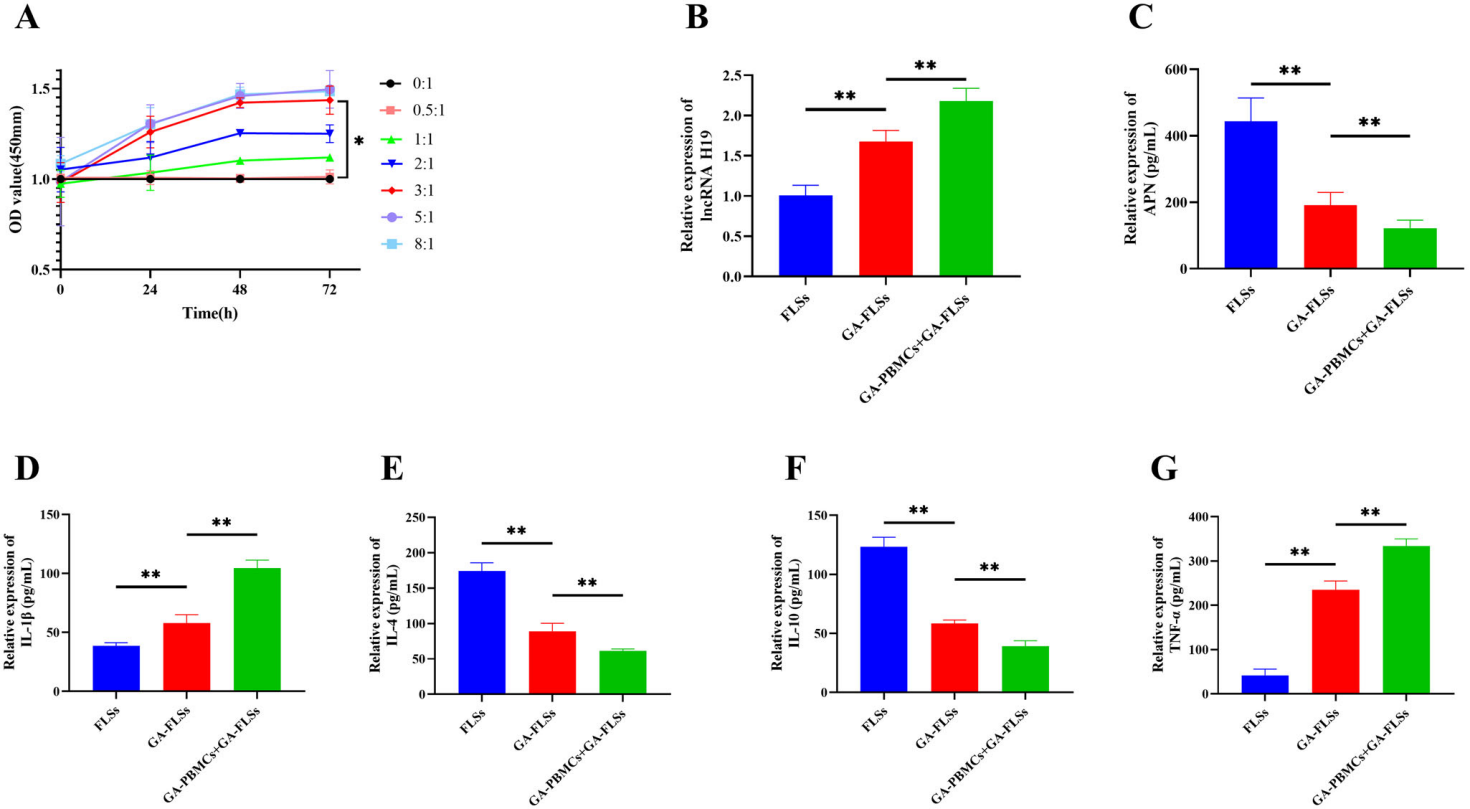
(A) Screening of co-culture concentration and time. Seven co-culture ratios of gouty arthritis peripheral blood mononuclear cells (GA-PBMCs) and gouty arthritis fibroblast-like synoviocytes (GA-FLSs) were set: 0:1, 0.5:1, 1:1, 2:1, 3:1, 5:1, and 8:1. The cell viability of GA-FLSs was detected by cell counting kit-8 assay at 0, 24, 48, and 72 h. At 24, 48, and 72 h, the cell viability of the 3:1, 5:1, and 8:1 groups was increased significantly compared with the 0:1, 0.5:1, 1:1, and 2:1 groups (*p* < 0.05), and there was no significant difference among the 3:1, 5:1, and 8:1 groups (*p* > 0.05). The cell viability of the 3:1 group was significantly increased at 48 h and 72 h compared with 0 h and 24 h (*p* < 0.05), while there was no significant difference in cell viability between 48 h and 72 h (*p* > 0.05). (B) The expression of long non-coding (lnc) RNA H19 was detected by real-time quantitative polymerase chain reaction. (C–G) The expression of adiponectin (APN), interleukin-1 beta (IL-1β), interleukin-4 (IL-4), interleukin-10 (IL-10), and tumor necrosis factor-alpha (TNF-α) was detected by enzyme-linked immunosorbent assay. *p < 0.05, **p < 0.01.

We set seven ratios (0:1, 0.5:1, 1:1, 2:1, 3:1, 5:1, and 8:1) in the co-culture of GA-PBMCs and GA-FLSs. The viability of FLSs was detected at 0, 24, 48, and 72 h, respectively. The results showed no significant difference in cell viability between the seven groups at 0 h (*p*** **>** **0.05). At 24, 48, and 72 h, the 3:1, 5:1, and 8:1 ratio groups showed significantly enhanced cell viability compared with the 0:1, 0.5:1, 1:1, and 2:1 ratio groups (*p*** **<** **0.05), but there was no significant difference in cell viability among the 3:1, 5:1, and 8:1 ratio groups (*p*** **>** **0.05). In addition, the cell viability of the 3:1 group was significantly increased at 48 h and 72 h compared with that at 0 h and 24 h (*p*** **<** **0.05), while there was no significant difference in cell viability between 48 h and 72 h (*p*** **>** **0.05) (Figure 7(A)). Finally, the optimal co-culture ratio of GA-PBMCs and GA-FLSs was determined to be 3:1, and the optimal reaction time was 48 h. Therefore, we chose a 3:1 culture ratio and 48 h culture time for the following experiments.

### Effect of GA-PBMCs on GA-FLSs

The expression of lncRNA H19, APN, and inflammatory cytokines in GA-FLSs before and after co-culture was detected. The results showed that the expression of lncRNA H19, IL-1β and TNF-α was significantly increased in GA-FLSs compared with those in FLSs, while the expression of APN, IL-4, and IL-10 was significantly decreased (*p*** **<** **0.01). The expression of lncRNA H19, IL-1β, and TNF-α in GA-FLSs stimulated by GA-PBMCs was significantly increased in comparison to GA-FLSs, while the expression of APN, IL-4, and IL-10 was significantly decreased (*p*** **<** **0.01) (Figure 7(B–G)). These results suggested that GA-FLSs presented with an increase in lncRNA H19, a decrease in APN, and an imbalance of inflammatory cytokines, and GA-PBMC stimulation aggravated lipid metabolism disorder and inflammation in GA-FLSs.

### Effect of lncRNA H19 on cell viability, APN, PI3K/AKT signaling pathway, and inflammatory cytokines

**Figure 8.**
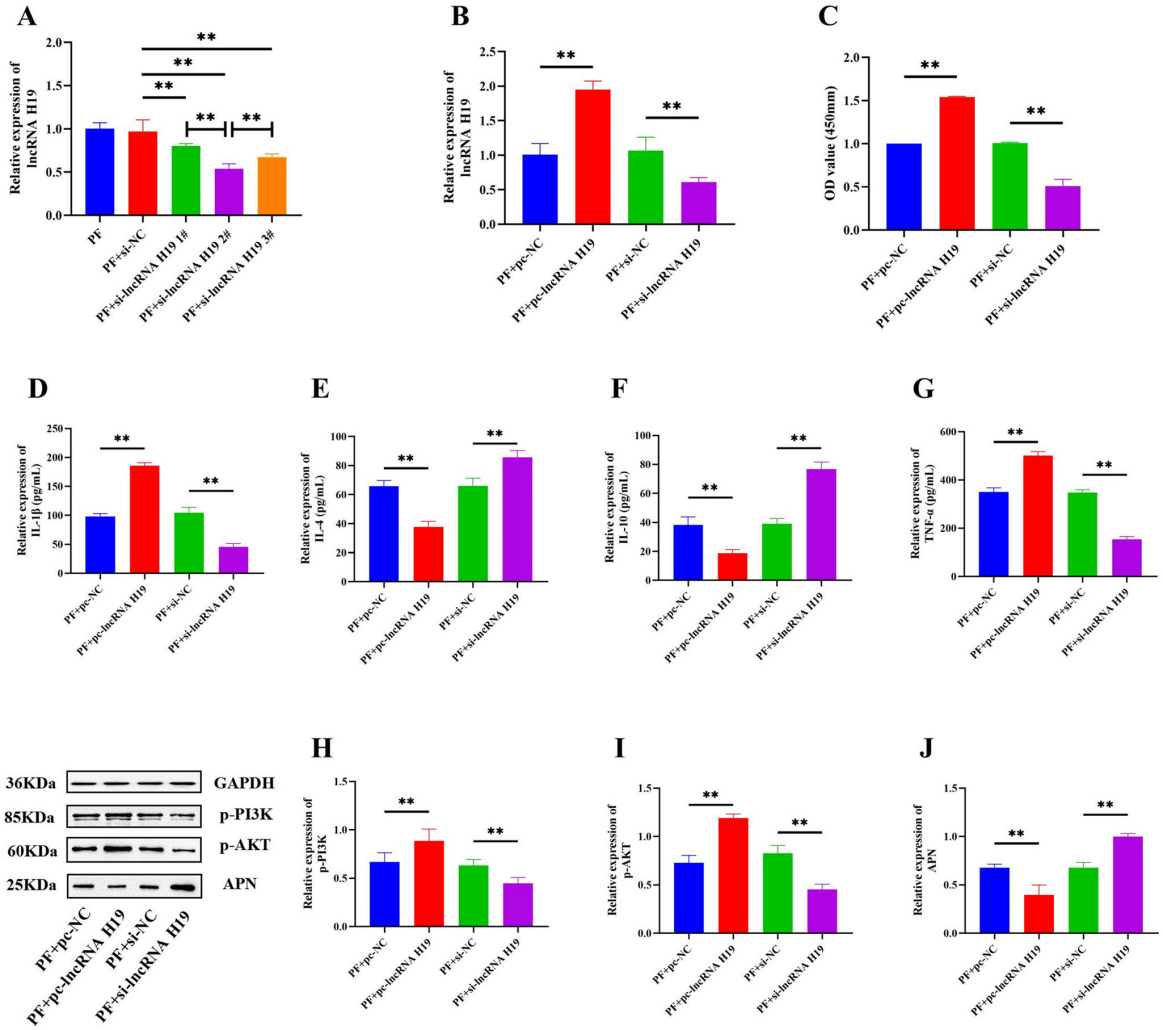
(A) Small interfering RNA screening of long non-coding (lnc) RNA H19. (B) The expression of lncRNA H19 was detected by real-time quantitative polymerase chain reaction in gouty arthritis fibroblast-like synoviocytes (GA-FLSs). (C) The cell viability of GA-FLSs was detected by cell counting kit-8 assay. (D–G) The expressions of interleukin-1 beta (IL-1β), interleukin-4 (IL-4), interleukin-10 (IL-10), and tumor necrosis factor-alpha (TNF-α) were detected by enzyme-linked immunosorbent assay in GA-FLSs. (H–J) The expression of p-PI3K, p-AKT, and adiponectin (APN) was detected by western blot in GA-FLSs. PF, gouty arthritis peripheral blood mononuclear cells + gouty arthritis fibroblast-like synoviocytes. **p < 0.01.

We constructed three siRNAs to knock down the expression of lncRNA H19, and the si-lncRNA H19 2# with the best transfection efficiency (*p*** **<** **0.01) was selected for subsequent experiments (Figure 8(A)). After the transfection efficiency validity of pc-lncRNA H19 and si-lncRNA H19, a CCK-8 assay was conducted and the results showed that compared with pc-NC or si-NC treatment, overexpression of lncRNA H19 enhanced the viability of GA-FLSs, while interference of lncRNA H19 attenuated cell viability (*p*** **<** **0.01) (Figure 8(B,C)). ELISA results showed that IL-1β and TNF-α were significantly up-regulated, while IL-4 and IL-10 were significantly down-regulated after lncRNA H19 overexpression, but these trends were reversed when lncRNA H19 was interfered (*p*** **<** **0.01) (Figure 8(D–G)). Western blot results showed that overexpression of lncRNA H19 increased the expression of p-PI3K and p-AKT and decreased the expression of APN, while interference of lncRNA H19 led to the opposite results (*p*** **<** **0.01) (Figure 8(H–J)). These results suggested that lncRNA H19 acted as an upstream functional RNA to regulate the activation of the PI3K/AKT pathway and the release of APN and inflammatory cytokines.

### LncRNA H19/APN/PI3K/AKT regulated the viability of GA-FLSs and the release of inflammatory cytokines

**Figure 9.**
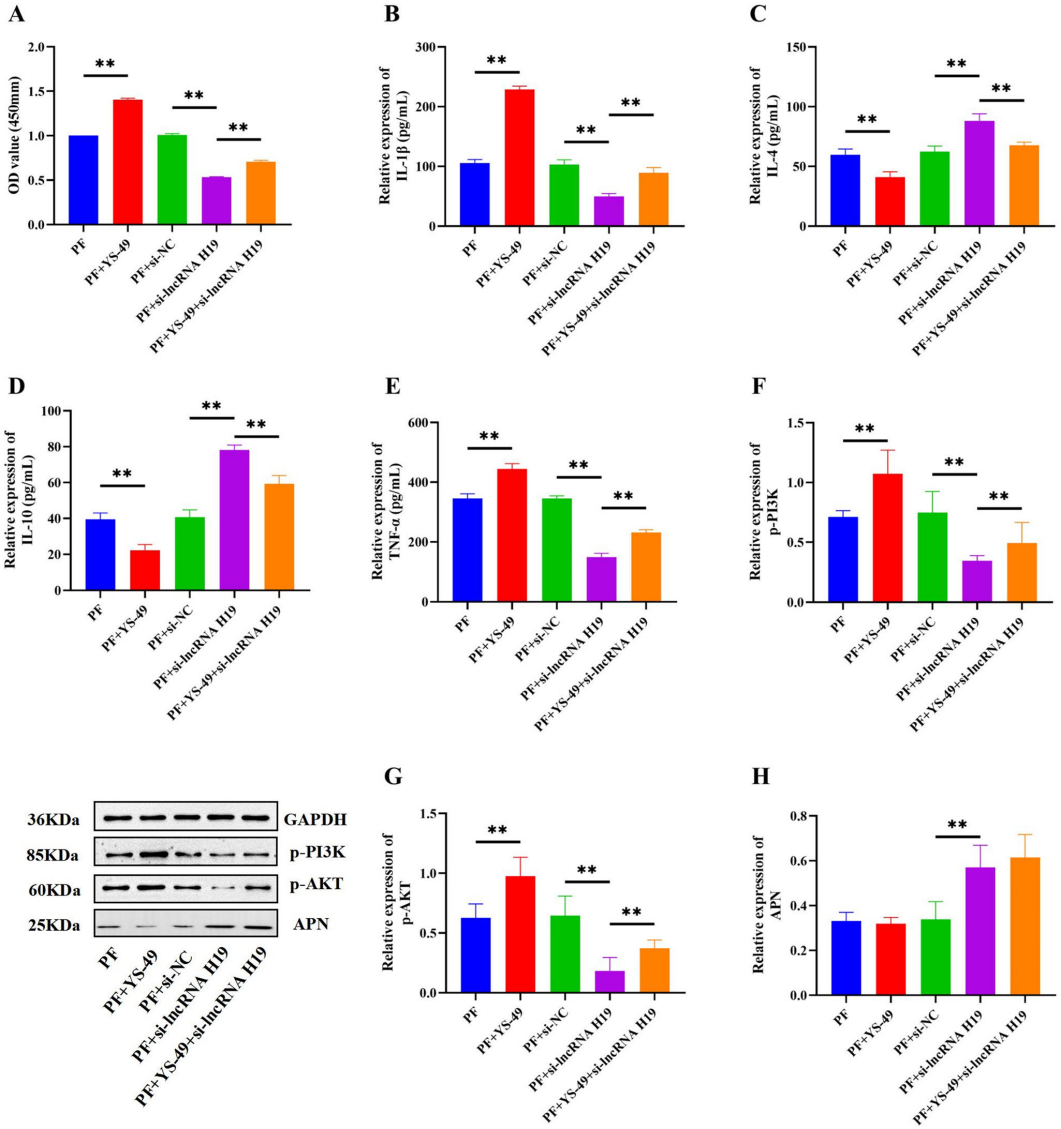
(A) The cell viability of gouty arthritis fibroblast-like synoviocytes (GA-FLSs) was detected by cell counting kit-8 assay. (B–E) The expression of interleukin-1 beta (IL-1β), interleukin-4 (IL-4), interleukin-10 (IL-10), and tumor necrosis factor-alpha (TNF-α) was detected by enzyme-linked immunosorbent assay in GA-FLSs. (F–H) The expression of p-PI3K, p-AKT, and adiponectin (APN) was detected by western blot in GA-FLSs. YS-49, an activator of the PI3K/AKT pathway. PF, gouty arthritis peripheral blood mononuclear cells + gouty arthritis fibroblast-like synoviocytes. **p < 0.01.

Although the above results confirmed that lncRNA H19 participated in the activation of the PI3K/AKT signaling pathway and the regulation of APN and inflammatory cytokines, the specific mechanism between PI3K/AKT, APN, and cytokines remained unclear. Therefore, we explored the interaction between them and detected the expressions of inflammatory cytokines after PI3K/AKT activation and APN inhibition. The results showed that the expressions of p-PI3K and p-AKT were significantly increased after the addition of the PI3K/AKT signaling pathway activator (*p*** **<** **0.01) (Figure 9(F,G)). Moreover, the expression of IL-4 and IL-10 was significantly decreased, but the expression of IL-1β and TNF-α was significantly up-regulated after PI3K/AKT activation (*p*** **<** **0.01) (Figure 9(B–E)). PI3K/AKT activation also enhanced the viability of GA-FLSs (*p*** **<** **0.01) (Figure 9(A)) but did not significantly affect the expression of APN (*p*** **>** **0.05) (Figure 9(H)). Subsequently, YS-49 was added after the interference of lncRNA H19, and the results showed that activation of the PI3K/AKT signaling pathway could partially reverse the decreased cell viability and anti-inflammatory cytokines and increased pro-inflammatory cytokines caused by lncRNA H19 interference (*p*** **<** **0.01) (Figure 9(A–E)), but it had no effect on the increase of APN (*p*** **>** **0.05) (Figure 9(H)).

**Figure 10.**
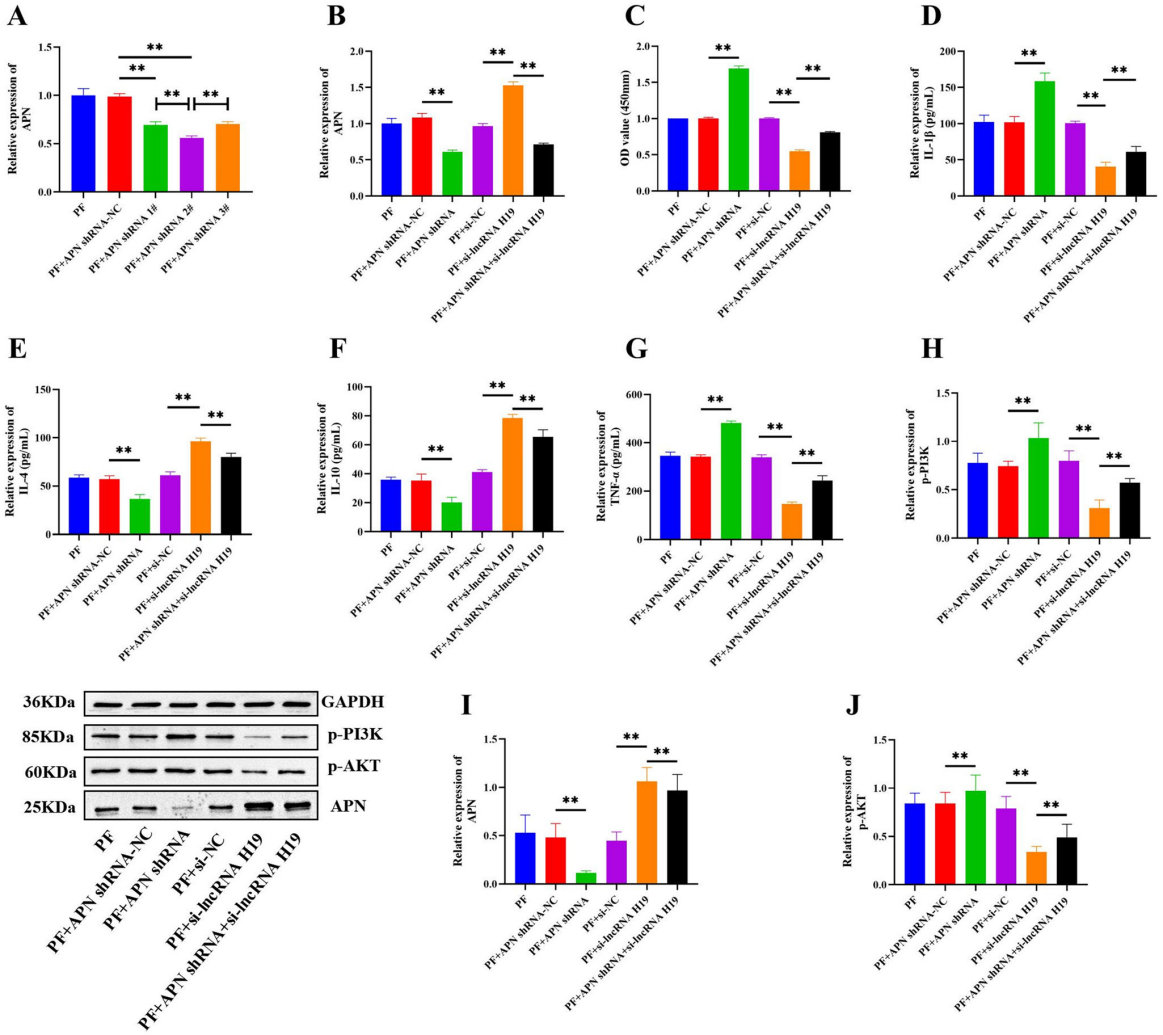
(A) Small hairpin RNA (shRNA) screening of adiponectin (APN). (B) The expression of APN was detected by real-time quantitative polymerase chain reaction in gouty arthritis fibroblast-like synoviocytes (GA-FLSs). (C) The cell viability of GA-FLSs was detected by cell counting kit-8 assay. (D–G) The expression of interleukin-1 beta (IL-1β), interleukin-4 (IL-4), interleukin-10 (IL-10), and tumor necrosis factor-alpha (TNF-α) was detected by enzyme-linked immunosorbent assay in GA-FLSs. (H–J) The expression of p-PI3K, p-AKT, and APN was detected by western blot in GA-FLSs. PF, gouty arthritis peripheral blood mononuclear cells + gouty arthritis fibroblast-like synoviocytes. **p < 0.01.

Then we constructed three APN shRNAs and found that the shRNA APN 2# had the highest transfection efficiency, so shRNA APN 2# was selected for subsequent experiments (*p*** **<** **0.01) (Figure 10(A)). Interference of APN significantly down-regulated the expression of APN (*p*** **<** **0.01) (Figure 10(B,I)), increased the expression of PI3K/AKT pathway-related proteins, IL-1β, and TNF-α (*p*** **<** **0.01) (Figure 10(D,H,J)), decreased the expression of IL-4 and IL-10 (*p*** **<** **0.01) (Figure 10(E–G)), and also significantly heightened the viability of GA-FLSs (*p*** **<** **0.01) (Figure 10(C)). We also interfered with APN after the interference of lncRNA H19 and found that the interference of APN could partially reverse the effect of lncRNA H19 interference on the PI3K/AKT signaling pathway, inflammatory cytokines, and cell viability (*p*** **<** **0.01) (Figure 10(B–J)). These results indicated an upstream and downstream regulatory relationship between lncRNA H19, APN, PI3K/AKT signaling pathway, and inflammatory cytokines. LncRNAH19 facilitated the activation of the PI3K/AKT signaling pathway by down-regulating APN, thus intensifying the viability and inflammatory response of GA-FLSs.

### HQC inhibited GA-FLSs inflammation via the LncRNA H19/APN/PI3K/AKT cascade

**Figure 11.**
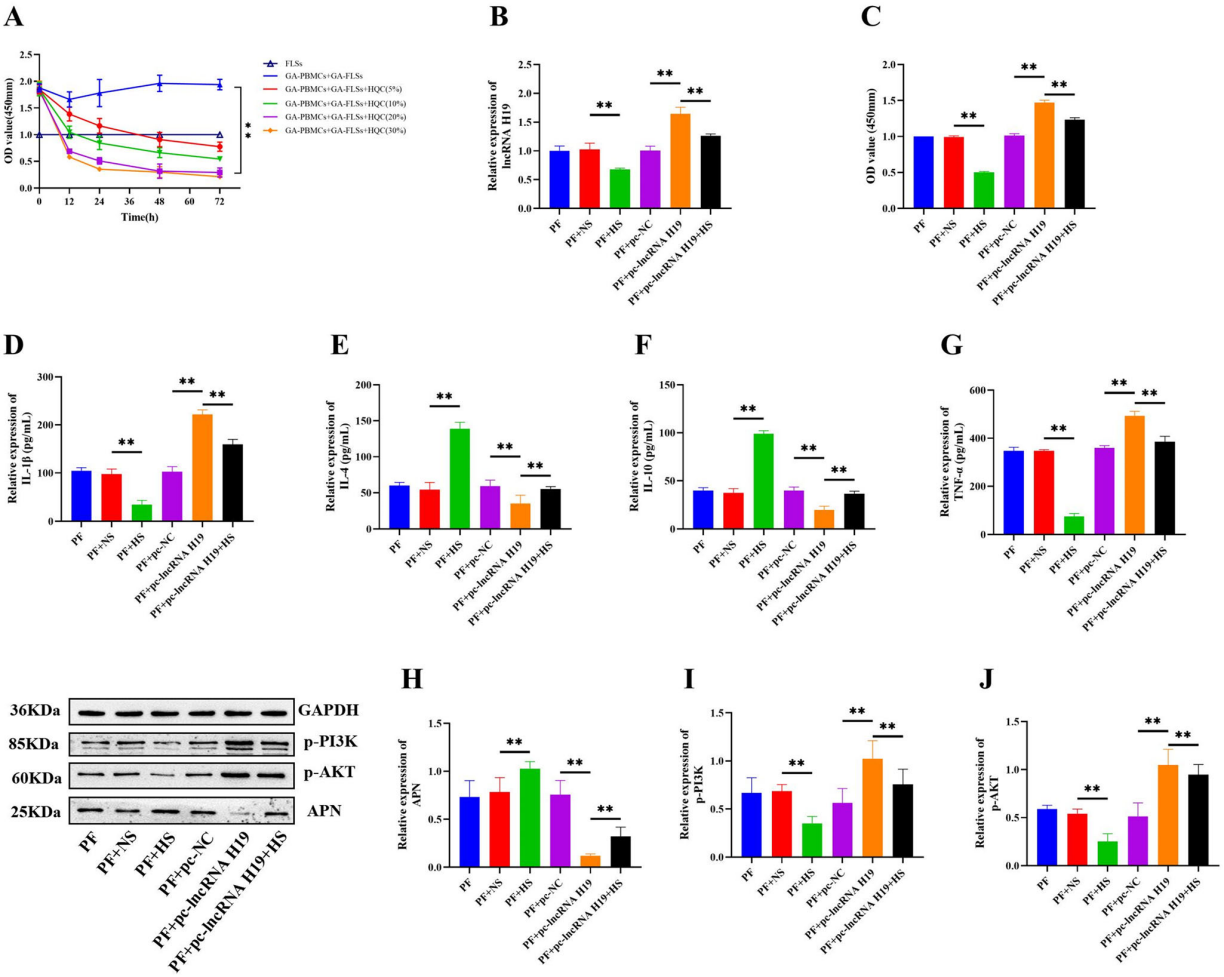
(A) Screening of Huangqin Qingrechubi capsule (HQC) drug-containing serum concentration. The cell viability of gouty arthritis fibroblast-like synoviocytes (GA-FLSs) was detected by cell counting kit-8 assay. Compared with the other groups, the cell viability of the 20% and 30% HQC drug-containing serum groups was significantly reduced (*p* < 0.01), but there was no significant difference in the cell viability between the 20% and 30% HQC drug-containing serum groups (*p* > 0.05). (B) The expression of long non-coding (lnc) RNA H19 was detected by real-time quantitative polymerase chain reaction in GA-FLSs. (C) The cell viability of GA-FLSs was detected by cell counting kit-8 assay. (D–G) The expression of interleukin-1 beta (IL-1β), interleukin-4 (IL-4), interleukin-10 (IL-10), and tumor necrosis factor-alpha (TNF-α) was detected by enzyme-linked immunosorbent assay in GA-FLSs. (H–J) The expression of p-PI3K, p-AKT, and APN was detected by western blot in GA-FLSs. PF, gouty arthritis peripheral blood mononuclear cells + gouty arthritis fibroblast-like synoviocytes. NS: normal serum; HS: HQC drug-containing serum. **p < 0.01.

To screen out the optimal concentration of HQC drug-containing serum, we set up medium containing 5%, 10%, 20%, and 30% HQC drug-containing serum for cell culture and detected the viability of GA-FLSs at 0, 12, 24, 48, and 72 h. The results showed that compared with that of the 5% and 10% HQC drug-containing serum groups, the cell viability of the 20% and 30% HQC drug-containing serum groups was significantly reduced (*p*** **<** **0.01), but there was no significant difference in cell viability between the 20% and 30% HQC drug-containing serum groups (*p*** **>** **0.05) (Figure 11(A)). Therefore, the 20% HQC drug-containing serum was selected for subsequent experiments. Compared with the PF (GA-PBMCs + GA-FLSs) group, the PF + NS (GA-PBMCs + GA-FLSs + Normal Serum) group had no significant changes in cell viability, inflammatory cytokines, lncRNA H19, APN, and PI3K/AKT pathway-related proteins (*p*** **>** **0.05) (Figure 11(B–J)), indicating that normal rat serum had no influence on the experimental results. Compared with the PF + NS group, the PF + HS (GA-PBMCs + GA-FLSs + HQC Drug-containing Serum) group had significantly up-regulated expression of IL-4, IL-10, and APN, but down-regulated expression of lncRNA H19, TNF-α, IL-1β, p-PI3K, and p-AKT and suppressed cell viability (*p*** **<** **0.01) (Figure 11(B–J)). Furthermore, HQC drug-containing serum was added after lncRNA H19 overexpression treatment, and the results showed that HQC drug-containing serum could partially reverse the effect of lncRNA H19 overexpression on APN, PI3K/AKT signaling pathway, inflammatory cytokines, and cell viability (*p*** **<** **0.01) (Figure 11(B–J)). These results suggested that HQC inhibited GA-FLSs viability and inflammatory response *via* the lncRNA H19/APN/PI3K/AKT cascade.

## Discussion

GA is one of the most common rheumatic diseases and leads to significant morbidity and disability, which not only causes substantial negative impact on the life of patients but also poses considerable burdens on clinical management (Butler et al. 2021). At present, the pathogenesis of GA has basically reached a consensus. GA occurs as a result of uric acid metabolism disturbance, in which urate crystals precipitate in the joint and elicit inflammatory responses, leading to swelling, redness, and pain of the joint (Ton and Kolber 2020). However, further studies suggest that the occurrence of GA is not only related to the imbalance of uric acid metabolism but other metabolic disorders such as abnormal lipid and glucose homeostasis should also be considered (Rasheed et al. 2014; Mu et al. 2020). Therefore, this study aims to determine whether metabolic disorders are involved in GA inflammation through clinical observation, network pharmacology, and *in vitro* cell experiments.

By comparing the clinical data of 30 GA patients and 30 healthy subjects, it was found that GA patients presented obvious disorders in inflammation, blood uric acid, and lipid metabolism. The expression of APN in the peripheral blood of GA patients was significantly reduced and significantly correlated with the expression of hs-CRP and IL-4. APN is an important lipid metabolism regulator that promotes lipid oxidation and increases lipid catabolism (Qiao et al. 2008). APN exhibits pro-inflammatory and anti-inflammatory heterogeneity in different diseases (Choi et al. 2020). In addition, APN data obtained by different researchers in the same disease are even contradictory, which makes the role of APN in inflammation controversial (Chen et al. 2022). In this study, we found that the expression of APN was reduced in the peripheral blood of GA patients, consistent with the results from a previous study (Sharonova et al. 2016). APN expression was found to be correlated with hs-CRP and IL-4, indicating the role of APN in inhibiting GA inflammatory response. Therefore, we speculated that APN mediated lipid metabolism and inflammatory response in GA and participated in the development of GA.

In the network pharmacology analysis, we obtained the target genes of active ingredients contained in HQC and collected the common target genes of GA, hyperlipidemia, and inflammation. After the intersection of the two groups of genes, we obtained the key genes of HQC acting on GA inflammation and lipid metabolism. Through PPI network construction and topology analysis, 30 key proteins including AKT1 were obtained. KEGG analysis showed that 14 of the 30 key proteins were enriched in the PI3K/AKT signaling pathway, ranking first except for the cancer-related pathway. It was suggested that the PI3K/AKT signaling pathway played a vital role in the improvement of GA inflammation and lipid metabolism by HQC. Therefore, we focused on the role of APN and PI3K/AKT in GA inflammation and lipid metabolism in subsequent cell experiments.

PBMCs constitute a critical part of the immune system, mainly including monocytes and lymphocytes. Lymphocytes represent principal cells of adaptive immunity as they circulate throughout blood into lymphoid tissues and initiate immune responses. The synovium is composed of matrix, cells, and fibers, which represents the main affected site of joint injury. Congenital immune synovitis of GA leads to synovial lesions, thus impairing joint functions (Liu et al. 2016). Hence, we used GA-PBMCs to stimulate GA-FLSs and established an *in vitro* cell model to simulate the *in vivo* state of GA patients. Our results showed that the expression of lncRNA H19 and pro-inflammatory cytokines in GA-FLSs was increased compared with those in FLSs, while the expression of APN and anti-inflammatory cytokines was decreased. After GA-PBMCs stimulation, such imbalance was deteriorated, further indicating the involvement of lncRNA H19 and APN in GA inflammation. Moreover, we conducted functional rescue experiments to validate the role of lncRNA H19 in GA inflammation. Our results confirmed that overexpression or inhibition of lncRNA H19 had significant impact on GA-FLSs viability, APN, p-PI3K, p-AKT, and inflammatory cytokines, indicating that lncRNA H19 was indeed involved in GA inflammation. Although lncRNA H19 has been reported to participate in the inflammatory development of arthritis by interacting with a variety of inflammatory pathways (Wang et al. 2020), the specific downstream mechanism of lncRNA H19 has not been confirmed. Therefore, we conducted APN inhibition and PI3K/AKT signaling pathway activation, respectively. The GA-FLSs viability, p-PI3K, p-AKT, and inflammatory cytokines were significantly changed after APN inhibition. Similarly, activation of the PI3K/AKT signaling pathway also significantly altered the GA-FLSs viability and the expression of inflammatory cytokines, while APN expression did not show significant changes. It was suggested that the regulatory relationship between APN and PI3K/AKT was unidirectional rather than bidirectional. Subsequently, we inhibited APN or activated PI3K/AKT signaling pathway after the interference of lncRNA H19, respectively. The results showed that inhibition of APN or activation of the PI3K/AKT signaling pathway could partially reverse the effect of lncRNA H19 interference, suggesting that overexpression of lncRNA H19 could activate the PI3K/AKT signaling pathway and promote the inflammatory progression of GA by inhibiting APN. It is well-established that the inflammation degree of GA is different in the stage of onset and remission. APN, as the most abundant adipokine in peripheral blood, act as an important regulator of lipid metabolism (Qiao et al. 2008). Accordingly, we speculated that APN exerted anti-inflammatory and anti-lipid effects in GA. GA is a metabolic disease, and urate deposition caused by uric acid metabolism disorder is a critical link in the pathogenesis of GA. However, uric acid metabolism disorder can also lead to lipid accumulation, and hypertrophic adipocytes further cause APN resistance and reduction (Engin 2017; Zhang et al. 2019; Chen et al. 2020). The reduction and resistance of APN can change the activity and efficiency of APN, and then, the joint inflammation caused by uric acid crystallization is aggravated after losing the inhibitory effect of APN on inflammation. It has also been reported that high levels of inflammatory reactants such as C-reactive protein may reduce the level of APN and make APN ineffective, thus forming a vicious cycle (Liu et al. 2016).

**Figure 12.**
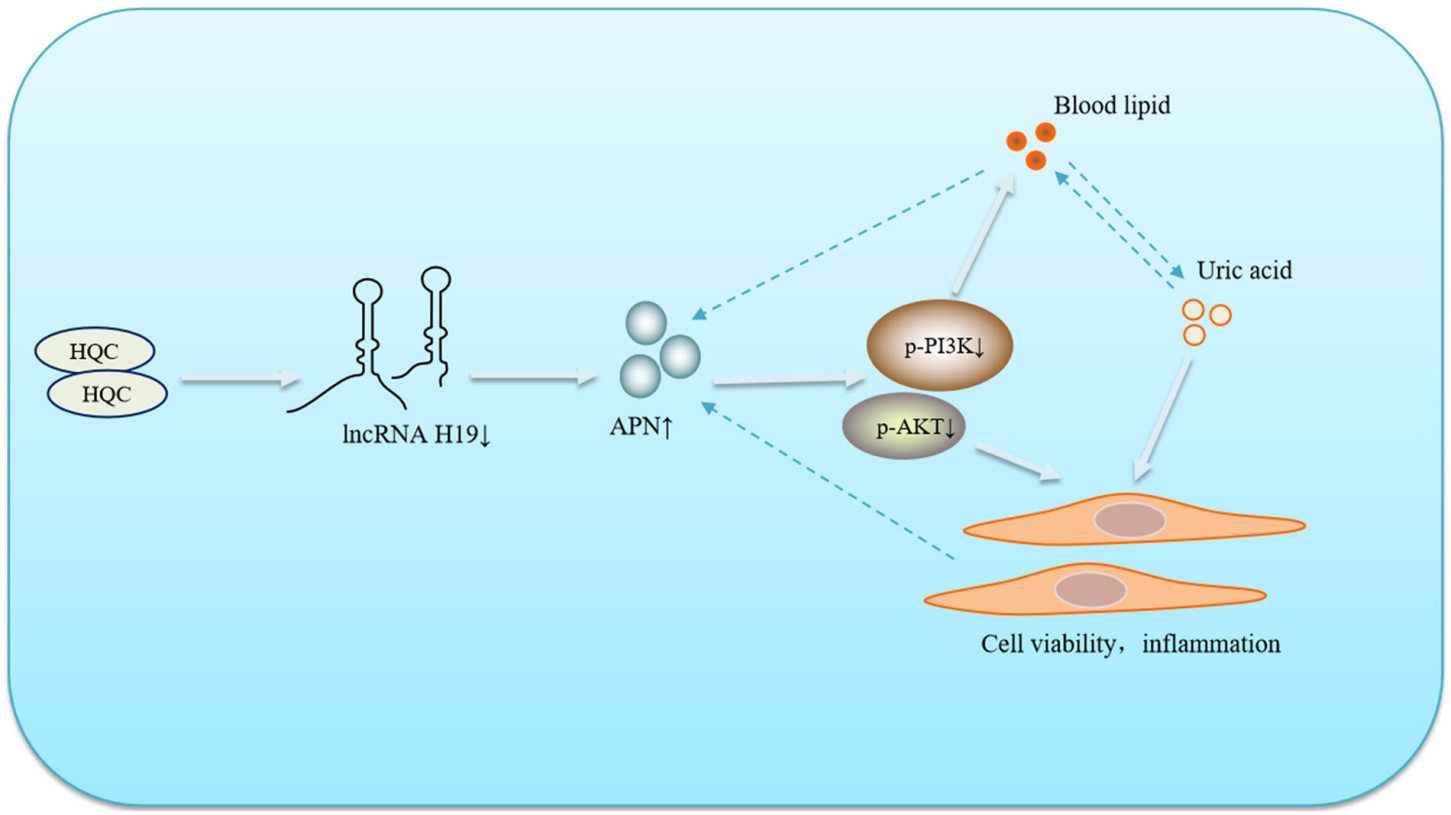
Huangqin Qingrechubi capsule (HQC) reduces the lipid metabolism disorder and inflammatory response in gouty arthritis *via* the lncRNAH19/APN/PI3K/AKT axis.

With the renovation of the world medical and health cause, Chinese herbal medicine and its pharmacologically active extracts have provided more treatment options for GA patients (Zhai et al. 2019; Duan et al. 2021; Zhai et al. 2021). HQC, a Chinese patent medicine prepared by the First Affiliated Hospital of Anhui University of Traditional Chinese Medicine, has shown favorable efficacy in the treatment of GA (Wang et al. 2014). In this study, our results revealed that HQC could down-regulate the expression of lncRNA H19, thereby affecting the APN/PI3K/AKT cascade and inhibiting the inflammatory response of GA-FLSs. Traditional Chinese medicine has always been characterized by multi-targets and multi-pathways in the treatment of diseases. In this study, the regulation of HQC on the key factor APN improved lipid metabolism disorder and inflammatory response in GA patients, interrupting the vicious cycle of lipid metabolism-APN-inflammation (Figure 12), which had certain reference significance for the development and use of drugs in the future. Since GA is a metabolic disorder, either uric acid metabolism disorder or glucose and lipid metabolism imbalance may promote the progress of GA. Therefore, uric acid metabolism, glucolipid metabolism, and inflammatory responses of GA should be fully considered in the clinical use of drugs.

In this study, the involvement of HQC in GA lipid metabolism and inflammation was explored through network pharmacology, clinical observation, and cell experiments, but our results failed to be verified *in vitro*. In future research, we will perform animal experiments to further validate the mechanism of HQC in regulating GA uric acid metabolism, lipid metabolism, and inflammation.

## Conclusions

Our findings elucidated that high expression of lncRNA H19 activated the PI3K/AKT signaling pathway and exacerbated lipid metabolism disorder and inflammation of GA by inhibiting APN. HQC treatment alleviated GA lipid metabolism disorder and inflammation *via* the lncRNA H19/APN/PI3K/AKT cascade, implying the promising potential of HQC in the treatment of GA.

## Data Availability

The datasets used and analyzed in the current study are available from the corresponding author on reasonable request.
